# High Frequency of Chronic Bacterial and Non-Inflammatory Prostatitis in Infertile Patients with Prostatitis Syndrome Plus Irritable Bowel Syndrome

**DOI:** 10.1371/journal.pone.0018647

**Published:** 2011-04-08

**Authors:** Enzo Vicari, Sandro La Vignera, Domenico Arcoria, Rosita Condorelli, Lucia O. Vicari, Roberto Castiglione, Andrea Mangiameli, Aldo E. Calogero

**Affiliations:** 1 Section of Endocrinology, Andrology and Internal Medicine, Department of Internal Medicine and Systemic Diseases, University of Catania, Catania, Italy; 2 Section of Gastroenterology, Department of Internal Medicine, University of Catania, Catania, Italy; Florida International University, United States of America

## Abstract

**Background:**

Although prostatitis syndrome (PS) and irritable bowel syndrome (IBS) are common disorders, information on the prevalence of IBS in infertile patients with PS is relatively scanty. Therefore, this study was undertaken to estimate the frequency of PS and IBS and to evaluate the prevalence of the various diagnostic categories of prostatitis.

**Methodology/Principal Findings:**

This study enrolled 152 patients with PS, diagnosed by the NIH-Chronic Prostatitis Symptom Index (NIH-CPSI) in an andrological setting, and 204 patients with IBS, diagnosed according to the Rome III diagnostic criteria in a gastroenterological setting. The patients with PS were asked to fulfill the Rome III questionnaire for IBS, whereas patients with IBS were asked to complete the NIH-CPSI. The simultaneous presence of PS and IBS was observed in 30.2% and 31.8% of the patients screened by andrologists and gastroenterologists, respectively. Altogether, 111 patients had PS plus IBS (31.2%). They had a total NIH-CPSI and pain subscale scores significantly higher than patients with PS alone. Gastrointestinal symptoms in patients with PS plus IBS were similar to those reported by patients with IBS alone and significantly greater in patients with PS alone. Patients with PS plus IBS had a significantly higher frequency of chronic bacterial prostatitis (category II) and lower of non-inflammatory prostatitis (category IIIB), compared to patients with PS alone. The frequency of inflammatory prostatitis (category IIIA) resulted similar.

**Conclusions/Significance:**

Prostatitis syndromes and IBS are frequently associated in patients with PS- or IBS-related symptoms. These patients have an increased prevalence of chronic bacterial and non-inflammatory prostatitis.

## Introduction

Prostatitis syndrome (PS) and irritable bowel syndrome (IBS) share some peculiar features: they are functional, somatoform disorders; have a high worldwide prevalence; symptoms have a substantially negative impact on the patients' quality of life; are defined on the basis of the clinical presentation rather than clear diagnostic markers or findings.

PS, a common syndrome affecting relatively young men, is the most frequent subtype of prostatitis encountered by family physicians, internists, and urologists. It has a prevalence of 11–16% [1], [2], an unclear etiology, and a significant negative impact on the quality of life, comparable to active Crohn's disease or a recent myocardial infarction [3]. The diagnosis of PS is based on the presence of chronic and variable symptoms mix which includes genitourinary pain (perineum, pelvis, suprapubic area and/or the external genitalia), the hallmark symptom of this syndrome. It causes also a variable degree of voiding and/or ejaculatory disturbance [4], [5]. To facilitate history taking and to establish a more uniform standard, the National Institutes of Health (NIH), USA, collaborative panel proposed the NIH-Chronic Prostatitic Symptom Index (NIH-CPSI), considered the first validated tool for assessing symptom severity in PS, useful for quantifying signs and symptoms and their impact on a patient's quality of life [5], [6]. In addition, among all the laboratory test designed to localize bacteria and/or leucocytes in segmented urinary tract specimens, the gold standard is the four glasses test proposed by Meares and Stamey [7]. It consists of obtaining the following specimens for microscopy and microbiological culture: the first voided 5–10 ml urine (VB1), midstream urine (VB2), pure prostatic secretion expressed by prostatic massage (EPS) and the first voided 5–10 ml urine after prostatic massage (VB3). The presence of microorganism(s) and/or leukocytes or no microorganisms and leukocytes in EPS and/or VB3 allows to diagnose chronic bacterial prostatitis (category II), inflammatory prostatitis (category IIIA) and non-inflammatory prostatitis (category IIIB), respectively [8]. Since bacterial prostatitis (acute or chronic) accounts for a low percentage (5–10%), the most significant proportion of the urologic population studied has chronic non-bacterial prostatitis (60–65%) or prostatodynia (30%) [9].

IBS is a functional disorder that affects 10–20% of the population worldwide, varying from 6.2% in The Netherlands to 12% in Italy and the UK [10], [11]. Its incidence is 1–2% per year [12]. IBS is defined by the Rome III criteria as an abdominal pain or discomfort, often associated with defecation (which relieves the pain), and at least two of the following features: altered frequency, consistency, and/or passage of stools; feelings of abdominal distension or bloating [12]. The symptoms have to be present for a minimum of three months, and evidence for an organic underlying cause must be excluded to establish the diagnosis [10]–[13]. The etiopathogenesis, which is not fully understood, may be multifactorial, as it is the pathophysiology, which is attributed to alterations of the gastrointestinal motility, visceral hypersensitivity, dysfunction of the brain-gut axis (abnormal interactions between exacerbating factors and gastrointestinal function, as well as on interaction between different parts of our nervous system and the gastrointestinal tract), neuroimmune dysregulation, post-infectious inflammation or certain psychosocial factors [14]–[16]. The Rome III criteria, in conjunction with a careful anamnesis (medical, familiar, and medication assumption) and thorough physical examination, should be applied as part of the stepwise, symptom-based approach to diagnose IBS.

A large number of patients with IBS is additionally afflicted by other somatic intestinal and/or extraintestinal co-morbidities [17], [18]. An elevated percentage of patients with IBS has other somatoform pain and psychiatric syndromes [19]–[22]. It is not clear whether the increase in frequency of extra-intestinal symptoms reflects a systemic underlying disease with different manifestations in various organs or it is expression of a psychosomatic disorder [23]. The best documented non-gastrointestinal, non-psychiatric disorders affecting IBS patients include: temporo-mandibular joint disorder (64%), chronic fatigue syndrome (51%), chronic pelvic pain (50%), and fibromyalgia (49%) [24].

Little is known about the possible presence of co-morbidities in patients with PS. Some of the most basic information are not available. For example, population-based, age-specific prevalence and incidence rates for many major urologic diseases are not known. Overlap between symptoms of different urologic conditions [eg, interstitial cystitis and chronic pelvic pain syndrome (CPPS), or CPPS and prostatitis] has not been adequately assessed. Only limited population-based information is presently accessible on the epidemiology of major urologic diseases. A smaller proportion is reported, receives an accurate diagnosis, and is recorded as a case, constituting only the “tip of the iceberg”. This epidemiological metaphor appears useful to understand urologic symptoms in the general population.

Recently, the research has focused on the co-morbidity between urological and non-urological unexplained clinical conditions, revealing a strong overlap up to 79% between chronic prostatitis (CP)/CPPS) and symptoms of IBS [25]. Furthermore, studies conducted in women showed allergies, urinary incontinence, sinusitis, endometriosis, pelvic inflammatory disease (PID), pelvic adhesions most frequently associated with chronic pelvic pain [26], [27]. In contrast, the few data available in men with CP/CPPS showed allergies, sinusitis, erectile dysfunction and IBS among the most common co-morbidities [28]. Li and co-workers found that the incidence of altered bowel habits was present in 31.4% of 379 men with chronic prostatitis [29].

Thus, this study was undertaken to evaluate the simultaneous presence of PS and IBS in two distinct groups of patients consulting an Andrology and a Gastroenterology clinical Divisions. To accomplish this, we enrolled 152 patients with PS, diagnosed by the NIH-Chronic Prostatitis Symptom Index (NIH-CPSI), and 204 patients with IBS, diagnosed according to the Rome III diagnostic criteria. The patients with PS were asked to fulfill the Rome III questionnaire for IBS, whereas patients with IBS were asked to complete the NIH-CPSI. In patients with PS plus IBS, we then evaluated the prevalence of the various diagnostic categories of prostatitis.

## Results

Of the 152 infertile patients with prostatitis diagnosed by andrologists, 46 (30.3%) had also IBS, whereas 65 (31.9%) out of 204 patients with IBS diagnosed by gastroenterologists had also prostatitis, with a median NIH-CPSI score of 14 (range 11–21), and couple's infertility. All patients with PS and IBS (n = 111) and the matched-group of the 106 patients with PS alone were categorized to diagnose the various categories of prostatitis and underwent semen analysis.

The mean age was similar in the patients with PS plus IBS, PS alone and in the group of fertile normal men (controls), whereas the time since diagnosis was higher, though not statistically significant, in patients with PS plus IBS (Table 1). Patients with PS plus IBS has a significantly higher total or pain subscale NIH-CPSI score compared with all the others groups of patients and controls (Table 1). The prevalence of various gastrointestinal symptoms was significantly higher in both groups of patients with PS plus IBS and IBS alone (Table 1). The severity score of abdominal bowel habits was similar in patients with PS plus IBS and IBS alone, but significantly higher than that found patients with PS alone or in the controls (Table 2).

**Table 1:** Demographic and symptoms of infertile patients with prostatitis syndromes (PS) and/or IBS and in a group of fertile men (controls).

Characteristics	PS plus IBS	PS alone	IBS alone	Fertile men
Number of patients	111	106	139	25
Age (years)	34±8.9	33±9.2	32±7.5	32±6.3
Time since diagnosis (years)	4±1.3	3±1.4	3±1.6	NA
**Prostatitis symptoms (NIH-CPSI score)**				
Total score	24±4.5*	18±3.8	11±3.5	3.2±1.0
Pain subscale	14.9±1.8*	10.2±3.0	8.8±2.6	0.8±0.3
Urinary subscale	4.8±1.7	4.5±1.9	3.0±1.7	1.1±0.5
Quality-of-Life subscale	7.2±1.7	6.2±1.8	6.0±1.8	1.4±0.2
**Gastrointestinal symptoms (frequency, %)**				
Abdominal pain/discomfort relieved by defecation	88.6±8.8†	17.2±6.2	82.7±10.7†	16±5.2
Change of bowel frequency	77.3±9.4†	24.1±6.3	75.5±9.8†	20±7.5
Change of fecal consistency	80.7±8.5†	25.8±6.6	76.2±8.9†	20±5.5
Constipation	51.1±8.6†	17.2±3.4	51.1±10.3†	16±2.7
Diarrhea	35.2±10†	8.6±4.8	41.0±2.2†	0
Bloating	56.8±11.5†	12.1±4.1	56.1±1.3†	8±1.0
Heartburn	50.0±10.3†	12.1±4.2	54.7±11.8†	8±1.0

IBS: irritable bowel syndrome; NA: not applicable.

Values are expressed as mean±SD.

*p<0.01 vs. the respective value observed in all the other groups of patients or in fertile men;

† p<0.01 vs. the respective value observed in the group with PS alone or fertile men.

**Table 2:** Severity score of abdominal bowel habits in infertile patients with prostatitis syndromes (PS) and/or IBS and in a group of fertile men (controls).

Symptoms score (mean, range)	PS plus IBS	PS alone	IBS alone	Fertile men
Number of patients	88	106	139	25
<3 bowel movements/week	1.6±1.14*	0.8±0.3	1.5±0.9*	0.6±0.1
>3 bowel movements/day	1.7±0.7*	0.7±0.2	2.0±1.2*	0.5±0.1
Hard or lumpy stools	1.8±1.1*	0.6±0.1	1.7±1.1*	0.4±0.1
Loose or watery stools	1.6±0.8*	0.6±0.2	1.6±1.2*	0.2±0.01
Defecation straining	2.2±0.4*	0.3±0.1	2.0±0.8*	0.2±0.01
Urgency	1.9±0.8*	0.7±0.3	1.8±0.9*	0.2±0.01
A feeling of incomplete bowel movement	2.4±1.5*	1.1±0.6	2.2±1.3*	0.5±0.1
Passing mucus	1.5±0.6*	0.7±0.1	1.4±0.8*	0.2±0.01
Abnormal fullness, bloating or swelling	3.0±1.5*	0.6±0.2	2.7±1.6*	0.4±0.2

IBS: irritable bowel syndrome.

Values are expressed as mean±SD;

*p<0.01 vs. the respective value observed in the group of patients with PS alone or in fertile men.

The subtyping of patients by using the four glass test, performed in all patients with PS plus IBS (n = 111) and in patients with PS alone (n = 106), allowed to identify the presence of 3 categories of prostatitis. The frequency of chronic bacterial prostatitis (category II) resulted significantly higher in patients with PS plus IBS compared to those with PS alone (p<0.01), whereas the frequency of non-inflammatory prostatitis (category IIIB) was significantly lower (p<0.01). Finally, the frequency of inflammatory prostatitis (category IIIA) was similar in the 2 groups (Table 3).

**Table 3:** Results from the Meares and Stamey test in infertile patients with prostatitis syndromes (PS) plus IBS or PS alone.

Findings in the expressed prostatic secretion (EPS) and/or in the voiding volume 3 (VB3)	PS plus IBS(n = 111, 100%)	PS alone(n = 106, 100%)	Types of prostatitis(NIH classification)
Microorganisms with or without leukocytes	30 (27.0%)*	15 (14.1%)	Chronic bacterial prostatitis (type II)
Leukocytes without microorganisms	54 (48.7%)	57 (53.8%)	Inflammatory prostatitis (Type IIIA)
No microorganisms and/or leukocytes in EPS	27 (24.3%)*	34 (32.1%)	Non-inflammatory prostatitis (Type IIIB)

IBS: irritable bowel syndrome.

*p<0.01 vs. the respective value in patients with PS alone.

## Discussion

It is worth of mention that while IBS diagnosis is based on an international consensus, such a consensus is missing for prostatitis syndromes. The second argument to keep in mind it that, in contrast to other syndromes that are usually based on a clear pathological model of the disease and well-defined clinical findings, IBS and PS symptoms are based on patients' subjective report of pain in the lower abdomen in the absence of organic explanations for it. Despite these limitations, the results of this joint andrological-gastroenterological study showed that the simultaneous presence of PS and IBS is very common being present in about one third of the patients evaluated and it had a similar prevalence in patients seeking medical advice for PS or IBS. This finding confirmed the epidemiological data showing a prevalence of altered bowel habits in the 31.4% of 379 patients with chronic prostatitis and the presence of prostate congestion and swelling closely related to altered bowel habits in these patients [29]. We observed that the gastrointestinal symptoms and the severity score was similar in patients with PS plus IBS versus patients with IBS alone. It is noteworthy that prostatitis symptoms, such as total score and pain subscale, were more likely to be diagnosed in patients with both diseases than in patients with PS or IBS alone.

Our study design, applied to patients with prostatitis symptoms and infertility, is corroborated by important results when the patients were subtyped for prostatitis category. Indeed, the four glass test [7], performed in all patients with PS plus IBS and in those with PS alone, allowed to discriminate three main conventional categories of prostatitis. The results showed that patients with PS plus IBS had a higher prevalence of chronic bacterial prostatitis and lower prevalence of non-inflammatory prostatitis compared to patients with PS alone. The prevalence of chronic bacterial prostatitis in this particular group of patients with the simultaneous presence of PS and IBS resulted higher than the low prevalence (5–10%) reported in other urological patient population. The other two types of prostatitis identified (categories IIIA and IIIB) had a similar prevalence: 60–65% for the category IIIA and 30% for the category IIIB [9]. On the other hand, the relative frequency of 14.1% in the PS group alone, apparently higher than in other publications, is comparable with that reported in our previous study showing that male accessory gland infections (MAGI), abnormal semen quality, abnormal ultrasound and no female infertility factor occurred in 122 out of 1187 (10.3%) infertile couples evaluated during a 6-year period [30]. Using the same criteria, this figure increased to 382 out of 2712 (13.8%) infertile couples taking into account all the infertile couples who came to our observation in the last 11 years.

Although the frequency of prostatitis in infertility is still unclear, the broad range of prevalence (1.6–15%) [31], [32] may be explained by the lack of a deeper diagnostic approach to the patient with not only PS but also infertility. The diagnosis PS should be completed looking for a clinical presentation of MAGI which more closely impair male fertility. Therefore, the wide range of prostatitis may reflects various factors, such as: a) different consulting physician speciality which has an impact on the work-up of the patient; b) incomplete and not well defined diagnosis (“sperm infection” is an improperly used laboratory word and it should be correctly replaced by the male diagnostic category defined conventionally as MAGI); c) lack of clinical characterization in patients initially fulfilling the conventional WHO criteria (clinical history, physical signs, and prostatic fluid and ejaculate signs). According to the hypothesis a), the elevated frequency of CBP reported in the present study may also reflect the fact that a large number of infertile patients, eventually to be submitted to assisted reproductive techniques, are referred to our Andrology Centre.

Finally, the higher prevalence of chronic bacterial prostatitis in patients with PS plus IBS compared with that found in patients with PS alone suggests to include ultrasound scans (didymo-epididymal and prostate-vesicular) in the diagnostic work-out of these patients to identify the possible presence of male accessory gland infection (MAGI) [33], [34] nested within this group. Indeed, we have previously reported that patients with prostate-vesiculo-epididymitis (PVE) have the worst sperm parameters and the highest number of seminal leukocytes and radical oxygen species production compared to patients with prostate-vesiculitus or prostatitis alone [32], [35] also in absence of important co-morbidities, such as IBS.

Our findings suggest that patients with PS plus IBS may have similar underlying pathophysiology, but they differ in severity. As several studies suggested that the pathophysiological mechanism of IBS are associated with specific symptom or subtype of prostatitis, we speculate that the some mechanisms that underlies the symptoms of IBS (such as an imbalance between commensal and pathogen bacteria of the intestinal microflora; local low-grade inflammation associated to IBS with abnormal immune function; intestinal motility; and the intraluminal milieu) may play a role in the pathophysiological mechanisms of both IBS and PS and particularly in the onset of chronic bacterial prostatitis. Since only 10–56% of adults with symptoms of IBS seek medical evaluation [36], [37], a joint gastroenterological and andrological counseling for men may represent a good opportunity in patients with PS. The presence of more severe prostatitis symptoms (such as a higher score at the pain subscale of the NIH-CPSI questionnaire) in patients with double morbidity (PS plus IBS) suggests to extend the application of the Rome III criteria to capture a possible presence of IBS in these patients. In the same way, the presence and/or the persistence of chronic bacterial prostatitis, even after the administration of antimicrobials and/or antinflammatory drugs, may suggest the presence of a conditioning co-morbidity such as the IBS.

In conclusion, this study showed that prostatitis syndromes and IBS are frequently associated in patients seeking medical advice for prostatitis- or IBS-related symptoms. Furthermore, the presence of IBS in patients with PS increases the prevalence of chronic bacterial prostatitis and non-inflammatory prostatitis. On this basis, we suggest that infertile patients with symptomatic PS should undergo also be explored for the presence of IBS.

## Materials and Methods

### Case selection

Men who had been seen by andrologists or gastroenterologists for symptoms that may be ascribed to PS or IBS, respectively. The protocol was approved by the internal Institutional Review Board and an informed written consent was obtained from each men.

#### Inclusion criteria

A patient was eligible for inclusion in this study if all of the following criteria applied: a) patient aged >21 years; b) presence of primary infertility, defined as lack of conception after at least 12 months of unprotected intercourse; c) symptoms suggestive of PS with an NIH-Chronic Prostatitic Symptom Index (NIH-CPSI) score >10; and d) diagnostic Rome III criteria for IBS.

#### Exclusion criteria

A patient was not eligible for inclusion in this trial if any the following criteria applied: a) history of disease with possible adverse effects on infertility (varicocele, cryptorchidism, sexual transmitted disease, testicular injury or torsion, environmental and occupational gonadotoxic exposure); b) presence of uni- or bilateral reduced testicular volume (<12 ml) at the physical examination; c) medical treatments in the previous 3 months; and d) known female factor (ovarian, tubal, cervical) of couple's infertility.

Twenty-five fertile men (mean age 35 yrs, range 32–45 yrs) who had fathered children within the previous 3 months and had attended our Andrology Centre for previous fertility counseling were also enrolled as controls. Fertile men were asked to complete the Rome III for IBS and the NIH-CPSI questionnaires for prostatitis.

### Study design and survey methods

#### Identification of IBS associated with PS

A group of 152 patients consulting the Andrology outpatient clinic of the University of Catania, mean age 33 years (range 26–45 years) with the diagnosis of symptomatic PS (NIH-CPSI score >10) were also asked to complete a Rome III questionnaire for IBS. Another group of 204 consecutive patients, consulting the Gastroenterology outpatient clinic of the University of Catania, mean age 34 years (range 28–45 years), diagnosed with IBS according to the Rome III diagnostic criteria and questionnaire for adult IBS, were also asked to fulfill the NIH-CPSI questionnaire for PS.

IBS patients were classified according to the frequency of nine different abnormal bowel habits (<3 bowel movements/week; >3 movements/day; hard or lumpy stools; loose or watery stools; straining during a bowel movements; urgency; feeling of incomplete bowel movements; passing mucus during a bowel movement; abdominal fullness, bloating or swelling) based on a 6-point scale (0, none; 1, <25% of the time; 2, 25–50% of the time; 3, 51–75% of the time; 4, >75% of the time, but not always; 5, always).

The association of two or more of the following symptoms, with recurrent abdominal pain or discomfort in at least 3 days/month in last 3 months, were considered IBS (criteria fulfilled for the last 3 months with symptom onset at least 6 months prior to diagnosis): 1) improvement with defecation; 2) onset associated with a change in frequency of stool; 3) onset associated with a change in form (consistency) of stool.

At the beginning of the study, all patients were evaluated by the same staff of andrologists, using a standardized history, physical examination, and symptom score evaluation with the NIH-CPSI questionnaire. Symptoms suggestive of PS included: pain or discomfort, for at least 3 months in the previous 6 months, in the pubic or bladder area, perineum, testicles, at the tip of the penis not related to urination; ejaculatory pain, pain or burning during urination, incomplete emptying, and urinary frequency. The NIH-CPSI is a validated scale to measure these symptoms [5], [6] which commonly uses 13-item for the assessment of symptom severity in men with PS, focusing on the three domains of: 1) pain or discomfort (8 items); 2) urinary symptoms (2 items); and 3) quality-of-life (QOL) impact (3 items). It has a total score range from 0 to 43, and includes 3 subscales addressing pain (score range 0–21), urinary symptoms (score range 0–10), and QOL (score range 0–12). The pain subscale consists of six items which are each scored from 0 to 1, one item which is scored from 0 to 5, and one item which is scored from 0 to 10. The urinary subscale consists of two items, each of which is scored from 0 to 3. The QoL subscale includes two items that are scored from 0 to 3, and one item that is scored from 0 to 6. More specifically, mild symptoms were defined by a pain score of 4–7 and moderate or severe symptoms by a pain score of ≥8. Symptoms and their effects were also grouped as mild, moderate and severe if CPSI scores ranged from 0 to 14, 15 to 30 and 31 to 43, respectively. The combined pain and urinary symptom score was then categorized into mild (0 to 9), moderate (10 to 16) and severe (17 to 31).

#### Categorization of prostatitis syndromes (PS)

Since the symptoms of chronic bacterial prostatitis (category II) cannot be distinguished from those of CP/CPPS (category IIIA and IIIB), following an initial screening, all patients with suspected PS (NIH-CPSI score >10) and IBS, underwent to the four glass test proposed by Meares and Stamey [7]. It requires that the following specimens will be obtained for microscopy and culture: the first voided 5–10 ml urine (VB1), midstream urine (VB2), pure prostatic secretion expressed by prostatic massage (EPS) and the first voided 5–10 ml urine after prostatic massage (VB3). The findings of microorganism(s) with or without leukocytes, leukocytes without microorganisms or no leukocytes and no microorganisms in EPS and/or VB3 suggests that diagnosis of chronic bacterial, inflammatory and non-inflammatory prostatitis, respectively [8]. The diagnosis of chronic prostatitis NIH type II was made clinically and based on microbiological tests following the consensus criteria of the NIH [8].

For the Meares and Stamey test, we considered the test positive if there was a 10-fold increase in bacteria in the EPS or in the VB3 samples compared with the VB1 and VB2 specimens. All the samples of this study were centrifuged and seeded on blood agar and McConkey media with standard biochemical tests to characterize bacteria. Microbiological evaluation included microscopic evaluation of VB1 with cytological analysis for leukocytes [38], quantitative culture methods for relevant pathogen bacteria (including Enterobacteriaceae, mycoplasmas, C. trachomatis and Candida spp.) in urethral discharge smears and in VB1 and either: C. trachomatis positive in VB1 and/or urethral smears, or presence of common bacteria, mycoplasmas, or yeasts with a concentration ≥10^4^ CFU/ml in the urethral discharge and/or ≥10^3^ CFU/ml in VB1, were considered criteria for diagnosis [38]. Additionally, inflammatory WBCs were counted on four-glass specimens and semen using the more stringent composite criteria (10+ in EPS, 5+ in VB3) described by Schaeffer and colleagues [39].

### Statistical Analysis

Results are shown as mean±SD throughout the text unless otherwise indicated. The Student's t test was used to compare the distribution of demographic, symptomatic and sperm variables. The frequency of clinical and microbiological findings between groups were analyzed by the chi-square test or Fisher's exact test, as appropriate. The sub-analysis of semen variables, because of the non-Gaussian distribution of the semen variables studied, were expressed as median and 10^th^ and 90^th^ percentiles and processed using non parametric tests (Mann-Whitney test). The software SPSS 9.0 for Windows was used for statistical evaluation. A statistically significant difference was accepted when the p value was lower than 0.05.
